# Supplementary material for: Flexible switch matrix addressable electrode arrays with organic electrochemical transistor and pn diode technology
Source: Nat Commun. 2024 Jan 15;15:533. doi: 10.1038/s41467-023-44024-1 (PMC10789794; doi:10.1038/s41467-023-44024-1)
Supplement: Supplementary file 1 — Supplementary information [file 41467_2023_44024_MOESM1_ESM.pdf]

Supplementary Figures for 'Flexible switch matrix addressable electrode arrays with organic electrochemical transistor and pn diode technology'

Ilke Uguz[1,#], David Ohayon[2], Volkan Arslan[1], Rajendar Sheelamanthula[3], Sophie Griggs[4], Adel Hama[2], John William Stanton[1], Iain McCulloch[3,4], Sahika Inal[2], Kenneth L. Shepard[1]

[1] Electrical Engineering Department, Columbia University, New York 10027, NY, U.S.A.

[2] Organic Bioelectronics Laboratory, Biological and Environmental Science and Engineering Division, King Abdullah University of Science and Technology (KAUST), Thuwal 23955-6900, Saudi Arabia.

[3] Physical Science and Engineering Division, KAUST, Thuwal 23955-6900, Saudi Arabia.

[4] Department of Chemistry, University of Oxford, Oxford OX1 3TA, United Kingdom.

Correspondence should be addressed to I.U. (email: ilkeuguz@gmail.com).

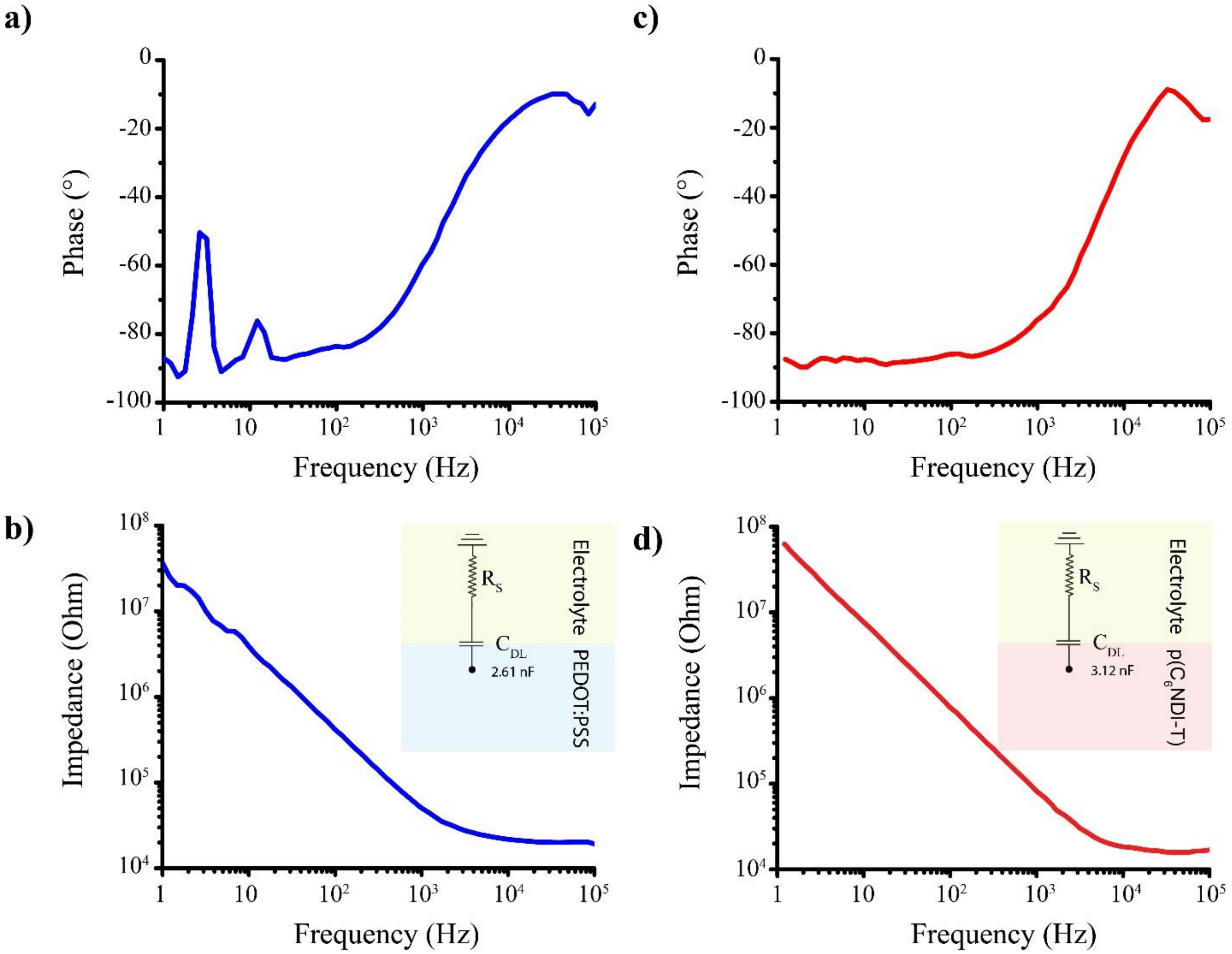

Supplementary Figure 1. Electrochemical impedance spectra of PEDOT:PSS and p(C_6NDI-T) films. Impedance **a)** phase and **b)** magnitude of the PEDOT:PSS electrode (150-nm film patterned on an area with a diameter of 20-µm) recorded at 1 to 100 kHz frequency range. Impedance **c)** phase and **d)** magnitude of the p(C_6NDI-T) electrode (150-nm film patterned on an area with a diameter of 20-µm) recorded at 1 to 100 kHz frequency range. (n=5 for each measurement.) Associated RC circuits are drawn at the inset with the double-layer interface capacitance value. The spectra were recorded in buffer electrolyte at open circuit potential (0 V vs. Ag/AgCl) for PEDOT:PSS and –0.4 V vs Ag/AgCl for p(C_6NDI-T).

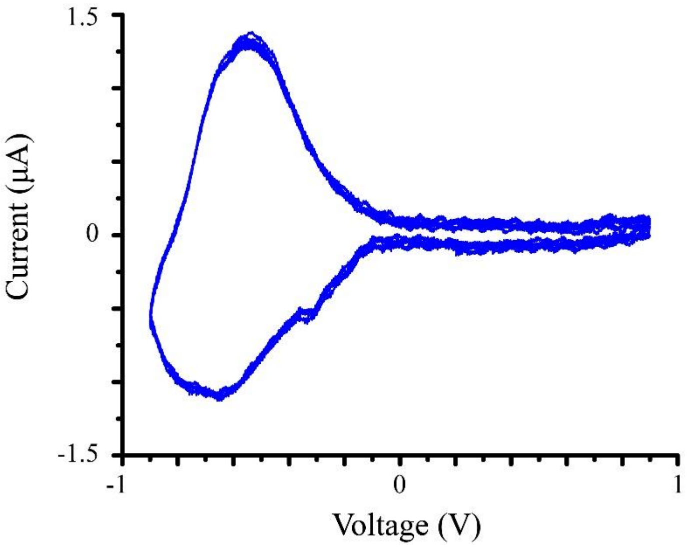

Supplementary Figure 2. Cyclic-voltammetry (CV) curve of p(C_6NDI-T) coated electrode. The Au electrode was coated with 150 nm of film on a 20 μm diameter pattern. The scan rate was 100 mV/s.

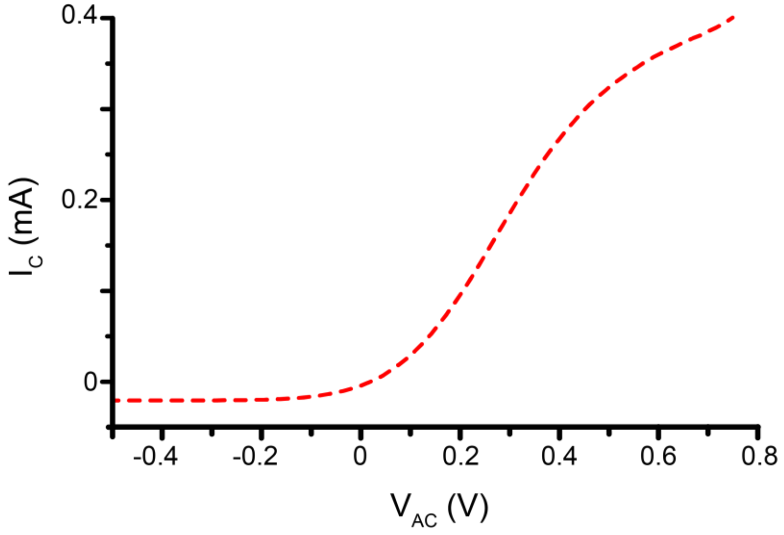

Supplementary Figure 3. Over-charged p-n OED current-voltage characteristics. Current response of the p-n OED corresponding to a 4 nC (generated with a V_{EC} of 1 V) injected charge.

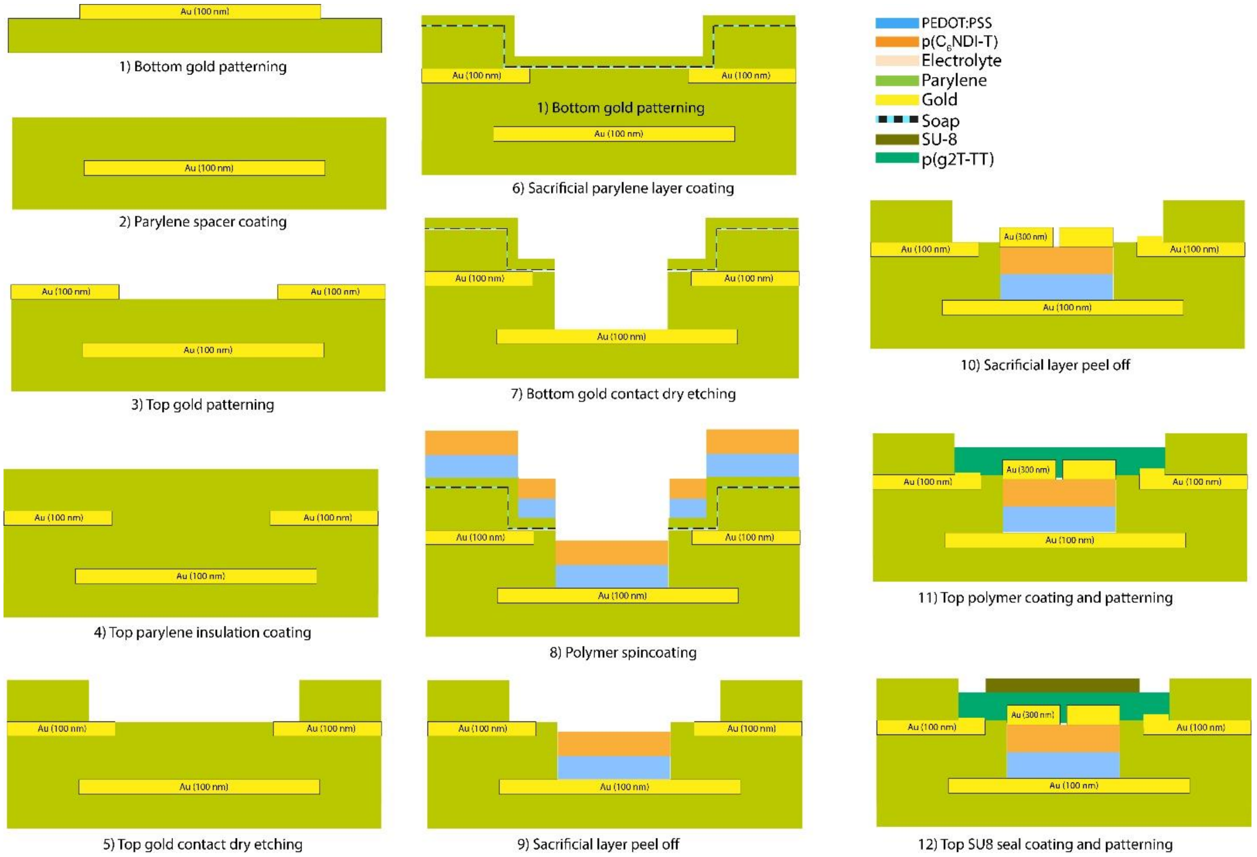

Supplementary Figure 4. Fabrication flow of the p-n OED. a) As cathode contact, 100 nm of Au is patterned on 2 µm layer of parylene-C carrier. **b)** A 0.8 µm layer of parylene-C is coated to provide insulation between vertical metal layers. **c)** Another 100 nm gold is patterned on top to define the source contact for subsequent OECT integration. **d)** A 2 µm layer is used as the top insulation layer. **e)** Top insulation is etched to access the top source contact. **f)** A 2 µm sacrificial layer of parylene-C is coated with soap underneath. **g)** Center parylene-C layer is etched through the hole inside the circular cathodic pad until the anode pad. **h)** The active materials are spin-coated sequentially to realize the vertical stack. **i)** Sacrificial layer of parylene-C is peeled off to complete polymer patterning. **j)** Top layer of Au is coated and patterned as the cathode contact. **k)** Top layer polymer (p(g2T-TT)) is coated and patterned. **l)** SU-8 seal is coated and patterned.

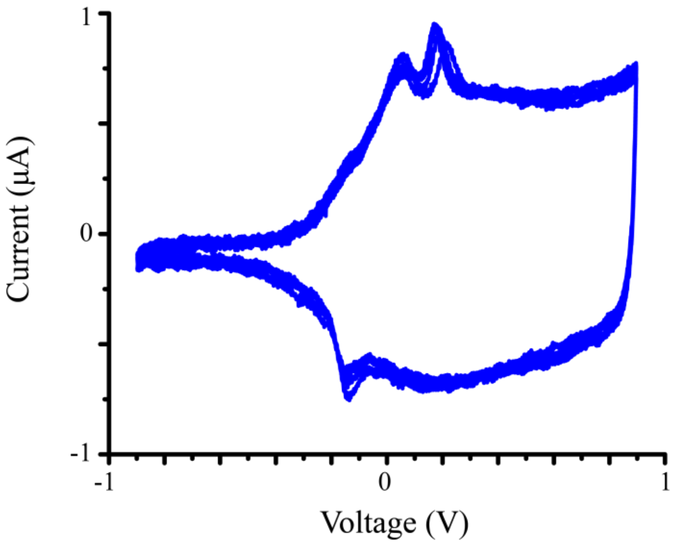

Supplementary Figure 5. CV curves of p(g2T-TT) coated electrode. The Au electrode was coated with 150-nm of film on a 20-μm diameter pattern. The scan rate was 100-mV/s.

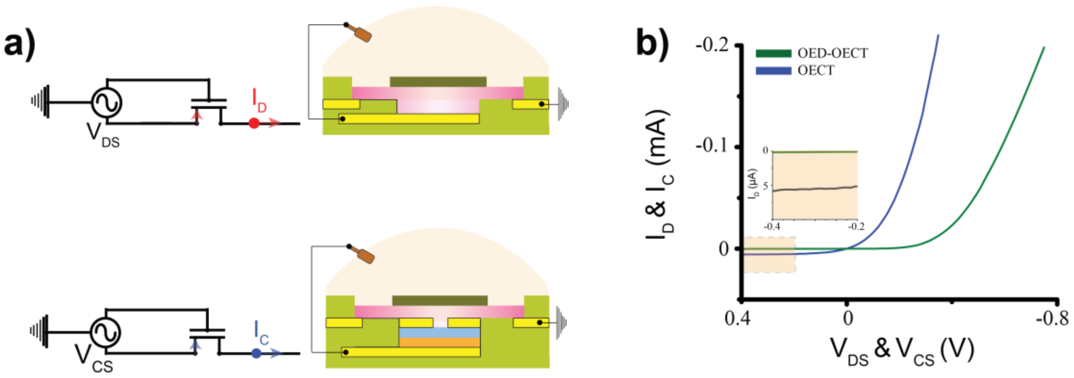

Supplementary Figure 6. Response characteristics of the single-element OECT and OED-OECT stack. a) OECT and OED-OECT stacks in diode-configured operation. A common voltage is applied to V_{DS} and V_{GS} (on top) for OECTs and a common voltage is applied to V_{CS} and V_{GS} (on bottom) for OED-OECT stacks. **b)** I-V characteristics of diode configured devices. The blue curve is the resulting I_D response of the OECT and the green curve is the resulting I_C response of the OED-OECT stack. (n=5 for each measurement.)

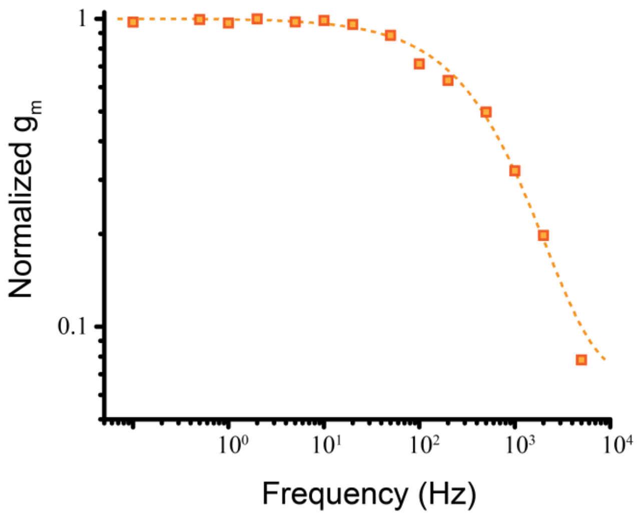

Supplementary Figure 7. Transconductance as a function of frequency. The OECT has a 250-nm thick channel and g_m (normalized to maximum g_m) is measured with a V_{GS} of -0.3 V and a V_{DS} of 0.3 V. (n=5 measurements.)

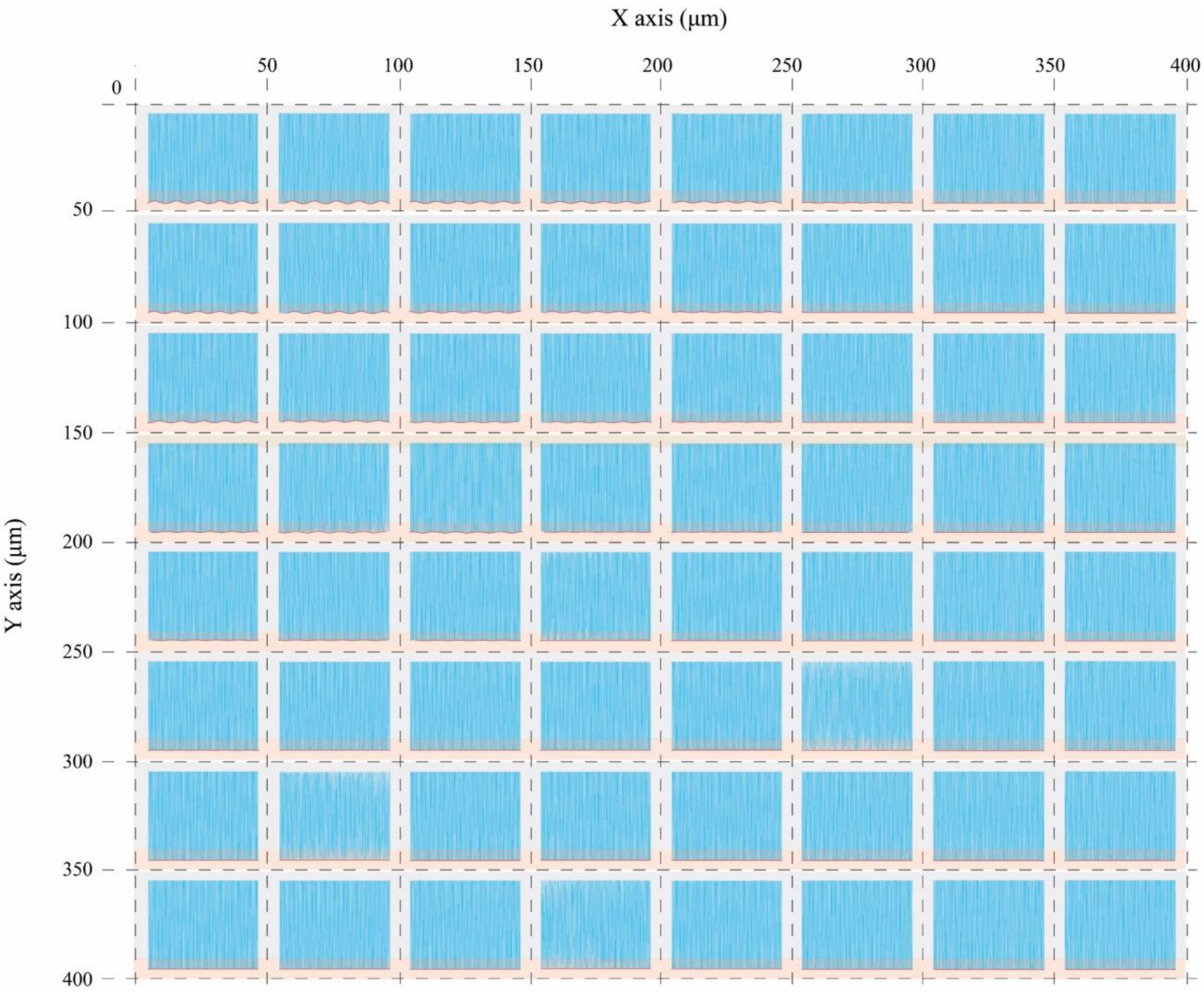

Supplementary Figure 8. 8x8 cross-point array recordings. The current output acquired from all individual OED-OECT stacks of an eight-by-eight cross-point array during applied stimulation pulses from an external electrode located at the top left channel. The OED-OECT stacks are operated separately with 1-ms pulses at 50% phase delay. The external pulses with 4-mV amplitude are applied at 10 Hz. The red highlights at the bottom of each graph represent the resulting waveforms.

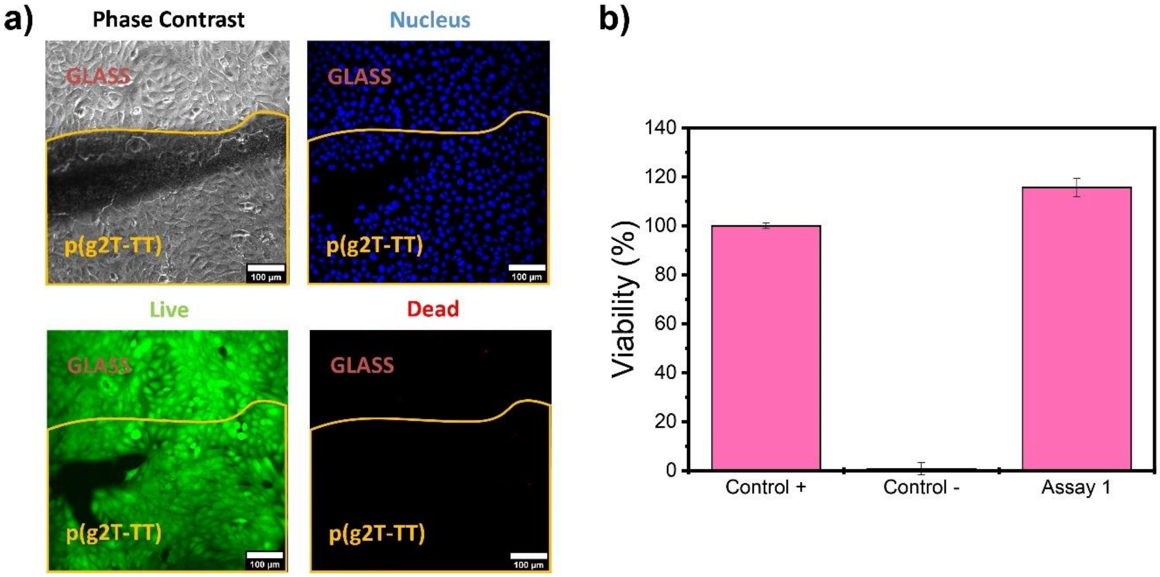

Supplementary Figure 9. Biocompatibility of the OED-OECT stack. a) Live-Dead Assay showing MCF10A cells growing after 48 hours in direct contact with p(g2T-TT). No dead cells are observed. **b)** Cytotoxicity assay of p(g2T-TT) films after 24 hours in conditioned media. Results do not show any cytotoxicity. Error bars represent the standard deviation (n=5).

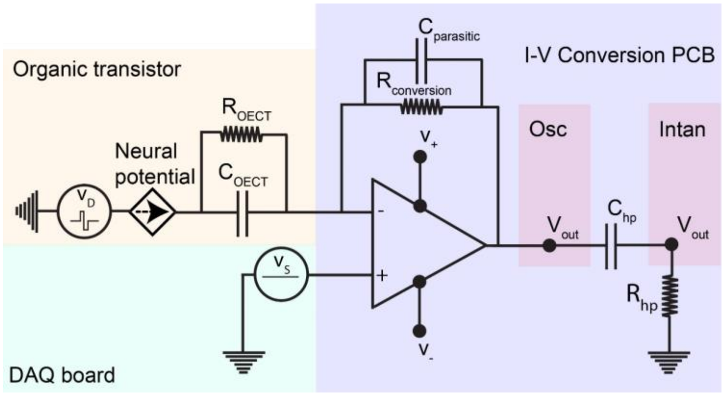

Supplementary Figure 10. Current-Voltage conversion for data acquisition *in vivo*. Sequential drain pulses are applied via a data acquisition board (Green). The current is run through the OECT, which is in contact with the electrolyte (Orange). A trans-impedance amplifier is used to convert the current to voltage. A high pass (0.15 Hz) and a parasitic low pass filters are used to minimize the noise (purple). Output of the amplifier is connected to an Oscilloscope or Intan head stage (following a high pass filter) for data acquisition (Red).

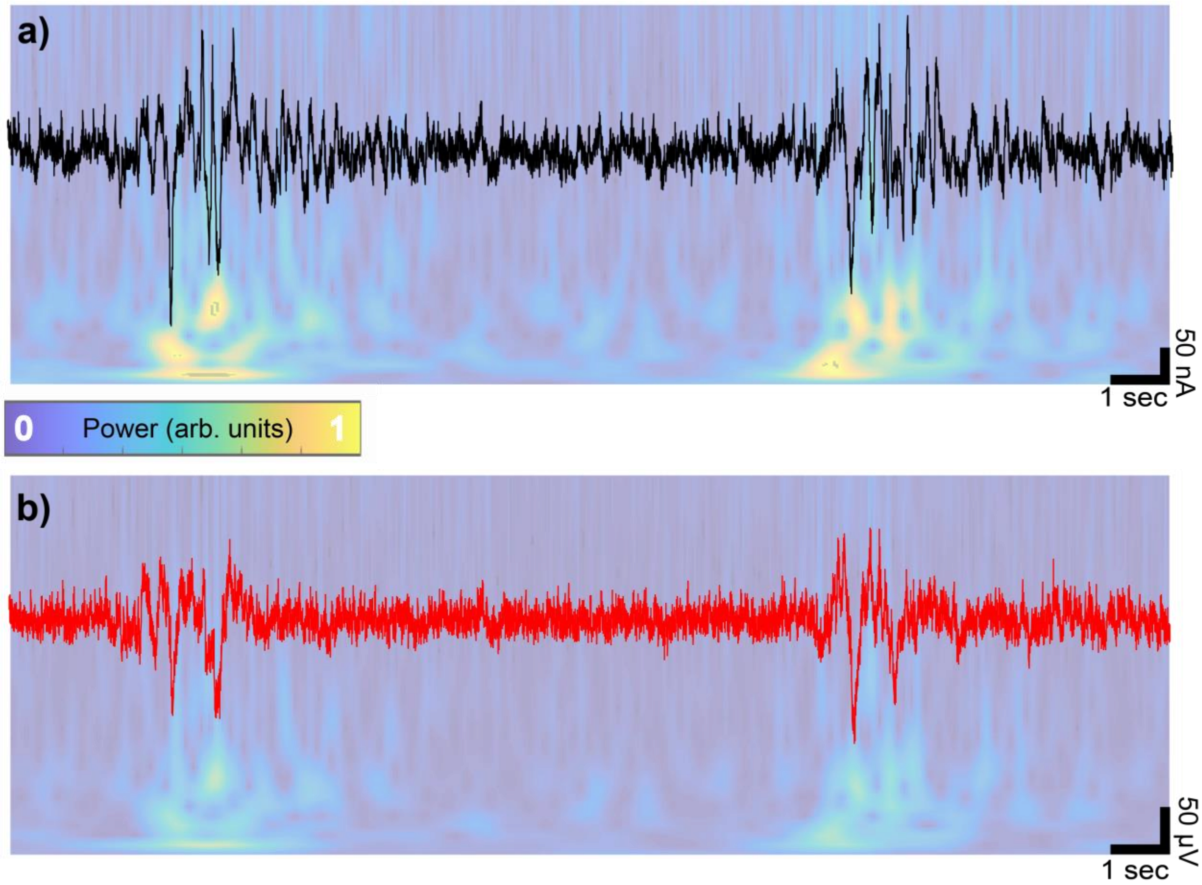

Supplementary Figure 11. Concurrent LFP recordings with active multiplexers and passive electrodes. Recordings from **a)** an OECT-OEDT stack and **b)** tungsten electrode located on the surface of the implantation zone. (n=3 implantations) The data are shown together with their corresponding time-frequency analysis plots. Time-frequency plots are within 0-40 Hz range and expressed in arbitrary units.

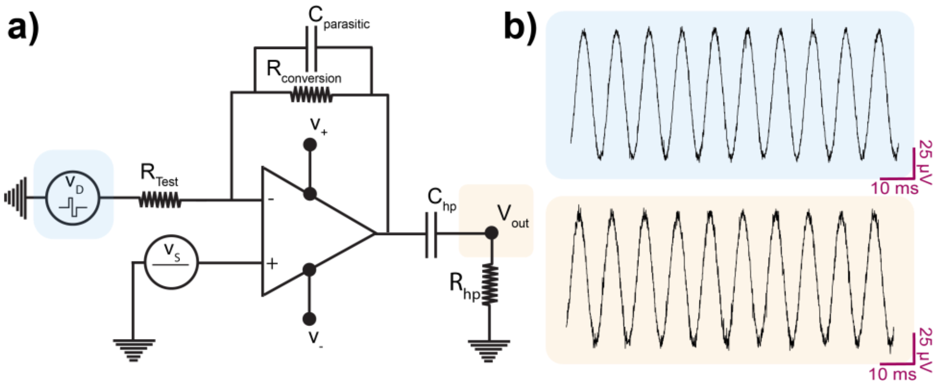

Supplementary Figure 12. Noise characteristics of the trans-impedance amplifier. a) Equivalent circuit model for noise analysis of the OED-OECT stack. **b)** The upper graph illustrates the V_D pulses applied by the drive electronics, while the lower graph displays the corresponding response observed in the output of the TIA.
