# Supplementary material for: Flexible switch matrix addressable electrode arrays with organic electrochemical transistor and pn diode technology
Source: Nat Commun. 2024 Jan 15;15:533. doi: 10.1038/s41467-023-44024-1 (PMC10789794; doi:10.1038/s41467-023-44024-1)
Supplement: Supplementary file 3 — Reporting Summary [file 41467_2023_44024_MOESM3_ESM.pdf]

nature portfolio

Corresponding author(s): Ilke Uguz

Last updated by author(s): Oct 26, 2023

Reporting Summary

Nature Portfolio wishes to improve the reproducibility of the work that we publish. This form provides structure for consistency and transparency in reporting. For further information on Nature Portfolio policies, see our Editorial Policies and the Editorial Policy Checklist.

Statistics

For all statistical analyses, confirm that the following items are present in the figure legend, table legend, main text, or Methods section.

n/a	Confirmed	
☐	☒	The exact sample size (n) for each experimental group/condition, given as a discrete number and unit of measurement
☐	☒	A statement on whether measurements were taken from distinct samples or whether the same sample was measured repeatedly
☒	☐	The statistical test(s) used AND whether they are one- or two-sided<br>*Only common tests should be described solely by name; describe more complex techniques in the Methods section.*
☒	☐	A description of all covariates tested
☒	☐	A description of any assumptions or corrections, such as tests of normality and adjustment for multiple comparisons
☐	☒	A full description of the statistical parameters including central tendency (e.g. means) or other basic estimates (e.g. regression coefficient) AND variation (e.g. standard deviation) or associated estimates of uncertainty (e.g. confidence intervals)
☒	☐	For null hypothesis testing, the test statistic (e.g. F, t, r) with confidence intervals, effect sizes, degrees of freedom and P value noted<br>*Give P values as exact values whenever suitable.*
☒	☐	For Bayesian analysis, information on the choice of priors and Markov chain Monte Carlo settings
☒	☐	For hierarchical and complex designs, identification of the appropriate level for tests and full reporting of outcomes
☒	☐	Estimates of effect sizes (e.g. Cohen's d, Pearson's r), indicating how they were calculated

Our web collection on statistics for biologists contains articles on many of the points above.

Software and code

Policy information about availability of computer code

Data collection	Intan RHS2000<br>Oscilloscope (DSO-X 4034A, Agilent technologies)<br>Autolab Potentiostat (Metrohm Autolab)<br>Semiconductor device analyzer (B1500A, Agilent Technologies)
Data analysis	MATLAB 2018b, OriginPro 8<br>Custom codes: github.com/klshepard/Electrophysiology-analysis-tools

For manuscripts utilizing custom algorithms or software that are central to the research but not yet described in published literature, software must be made available to editors and reviewers. We strongly encourage code deposition in a community repository (e.g. GitHub). See the Nature Portfolio guidelines for submitting code & software for further information.

Data

Policy information about availability of data

All manuscripts must include a data availability statement. This statement should provide the following information, where applicable:

- Accession codes, unique identifiers, or web links for publicly available datasets
- A description of any restrictions on data availability
- For clinical datasets or third party data, please ensure that the statement adheres to our policy

Any data collected in this study is presented in the Figures/Supplementary files. The source data are available as a Source Data file for the respective figures.

Human research participants

Policy information about studies involving human research participants and Sex and Gender in Research.

Reporting on sex and gender	NA
Population characteristics	NA
Recruitment	NA
Ethics oversight	NA

Note that full information on the approval of the study protocol must also be provided in the manuscript.

Field-specific reporting

Please select the one below that is the best fit for your research. If you are not sure, read the appropriate sections before making your selection.

[x] Life sciences [ ] Behavioural & social sciences [ ] Ecological, evolutionary & environmental sciences

For a reference copy of the document with all sections, see nature.com/documents/nr-reporting-summary-flat.pdf

Life sciences study design

All studies must disclose on these points even when the disclosure is negative.

Sample size	The figure legends contain information about the reported sample sizes for all cases.
Data exclusions	No data was excluded from the analysis.
Replication	Figure legends contain information regarding the number of replications for each measurement, and device.
Randomization	We did not perform randomization as it is not applicable to this study design.
Blinding	We did not perform blinding.

Reporting for specific materials, systems and methods

We require information from authors about some types of materials, experimental systems and methods used in many studies. Here, indicate whether each material, system or method listed is relevant to your study. If you are not sure if a list item applies to your research, read the appropriate section before selecting a response.

Materials & experimental systems

n/a	Involved in the study	
x		Antibodies
	x	Eukaryotic cell lines
x		Palaeontology and archaeology
	x	Animals and other organisms
x		Clinical data
x		Dual use research of concern

Methods

n/a	Involved in the study	
x		ChIP-seq
x		Flow cytometry
x		MRI-based neuroimaging

Eukaryotic cell lines

Policy information about cell lines and Sex and Gender in Research

Cell line source(s)	Breast epithelial MCF10A cell line, Vendor: ATCC (American Type Culture Collection)
Authentication	The cell line is not in the list of known misidentified cell lines maintained by the International Cell Line Authentication Committee Authentication Committee.
Mycoplasma contamination	Hoechst DNA stain (indirect) method<br>Agar culture (direct) method<br>PCR-based assay
Commonly misidentified lines<br>(See ICLAC register)	NA

Animals and other research organisms

Policy information about studies involving animals; ARRIVE guidelines recommended for reporting animal research, and Sex and Gender in Research

Laboratory animals	Male mice for this project were acquired from Jackson labs: Wild-type mice (000664) of 6-months age old were used for recordings. Housing conditions typically involve a 12-hour light and 12-hour dark cycle, with temperatures maintained in the range of 18-23°C, and humidity levels maintained in the 40-60% range.
Wild animals	No wild animals were used in this study.
Reporting on sex	Male mice were used in this study.
Field-collected samples	No field-collected samples were used in this study.
Ethics oversight	The Institutional Animal Care and Use Committee (IACUC) reviewed and approved protocols for Columbia University's program for the humane care and use of animals and inspects the animal facilities and investigator laboratories. Evaluation of the implanted devices was performed in compliance with Animal Welfare and Columbia's IACUC regulations under approved IACUC protocol.

Note that full information on the approval of the study protocol must also be provided in the manuscript.
